# Green and cost-effective voltammetric assay for spiramycin based on activated glassy carbon electrode and its applications to urine and milk samples†

**DOI:** 10.1039/d2ra06768d

**Published:** 2023-01-03

**Authors:** Hind A. M. Noureldin, Ali M. Abdel-Aziz, Mokhtar M. Mabrouk, Amira H. K. Saad, Ibrahim H. A. Badr

**Affiliations:** Department of Analytical Chemistry, Faculty of Pharmacy, Badr University in Cairo Cairo Egypt; Department of Chemistry, Faculty of Science, Ain Shams University Cairo Egypt ihbadr@sci.asu.edu.eg; Department of Analytical Chemistry, Faculty of Pharmacy, Tanta University Tanta Egypt; Department of Chemistry, Faculty of Science, Galala University Suez Egypt ibrahim.badr@gu.edu.eg

## Abstract

A simple, cost-effective, and efficient differential pulse voltammetric (DPV) assay for monitoring spiramycin adipate (SPA) in its dosage forms, urine, and milk samples at an activated glassy carbon electrode (GCE) was developed. GCE was electrochemically activated by anodization at a high positive voltage (2.5 V). The activated glassy carbon electrode (AGCE) was surface characterized, optimized, and utilized for the electrochemical assay of SPA. The electrochemical behavior of the AGCEs was investigated using cyclic voltammetry (CV) which shows a remarkable increase in the anodic peak of SPA in comparison with GCE. This behavior reflects a remarkable increase in the electrocatalytic oxidation of SPA at AGCE. The impacts of various parameters such as scan rate, accumulation time, and pH were investigated. The analytical performance of the activated glassy carbon electrodes was studied utilizing DPV. Under optimum conditions, the oxidation peak current exhibited two linear ranges of 80 nm to 0.8 μM and 0.85–300 μM with a lower limit of detection (LOD) of 20 nM. The developed assay exhibited high sensitivity, excellent repeatability, and good selectivity. Additionally, the developed SPA-sensitive modified GCE was successfully applied for SPA assay in its pharmaceutical dosage form and diluted biological fluids as well, with satisfactory recovery results which correlated well with the results obtained using spectrophotometry.

## Abstract 2

An electrochemically activated glassy carbon electrode was utilized as a cost-effective, environmentally friendly, and operationally simple sensor for sensitive and selective determination of spiramycin in dosage form, spiked milk and urine samples.

## Graphical abstract

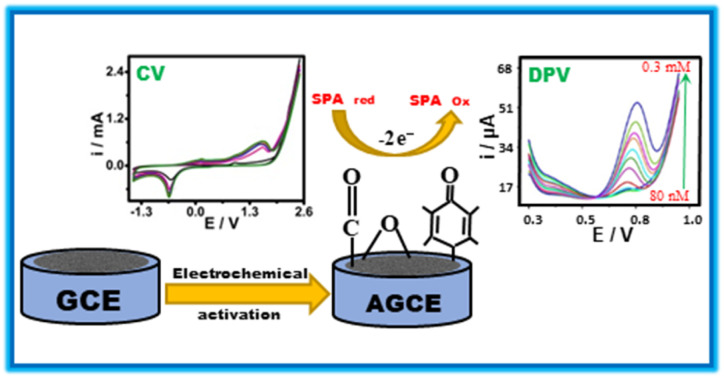

## Introduction

Most antibiotics of natural origin are derived from actinomycetes.^1,2^ Macrolides are among those antibiotics. Macrolides mainly consist of a lactone ring that differs in size (12 to 16 atoms).^3^ Macrolides are not only applied for human use but have also a wide range of veterinary uses.^4^ For instance, macrolides are used as feed additives to foster animal growth and reduce the incidence of animal disease and animal death.^5^ Spiramycin, a 16-membered macrolide antibiotic, is produced by the fermentation of *Streptomyces ambofaciens*.^6^ Spiramycin is also applied for human therapeutic use for the treatment of acute acquired toxoplasmosis in pregnancy by preventing the transmission to the fetus since it is concentrated in placenta tissue.^7^ Overuse of antibiotics could lead to their accumulation in food and water, which may result in severe threats to human health. Moreover, the systemic administration of many classes of antibiotics at sub-therapeutic doses may leave residues in the edible tissues of animals or food of animal origin (*e.g.*, meat and milk).^8^ The residues of those medications in food not only may lead to disorders in the public consumers^9^ but they may also induce problems and complications in the milk industry.^10^ Therefore, to guarantee superior quality of food and to keep human health safe, the European Union and the Swiss regulation authorities^11^ have established maximum residue limits (MRLs) of these antibiotics in food. The MRLs of spiramycin are 0.15 mg kg^−1^,^12^ and 0.2 ppm in milk and animal muscles,^13^ respectively.

Conventional instrumental analysis techniques have been utilized for the assay of SPA in dosage forms and real samples (*e.g.*, plasma and tissues) including and not limited to high-performance liquid chromatography (HPLC),^14–18^ spectrophotometry,^19^ liquid chromatography,^20^ electrophoresis,^21^ potentiometry,^22^ voltammetry,^23^ polarography,^24^ microbiological analysis,^25,26^ and radioactive assay.^27^ Furthermore, an assay of SPA level in milk samples was performed using a number of those analytical methods such as liquid chromatography with tandem mass spectrometry,^28,29^ micellar electrokinetic capillary chromatography,^30^ and HPLC equipped with fluorescence detection.^12^ Although those methods have various merits such as sensitivity and accuracy, the expensive instrument cost, tedious separation procedures, and laborious operations limit their extensive routine applications.^31^ Most of such methods (*e.g.*, HPLC) end up with organic waste that is hazardous for human health, which creates a need for eco-friendly and cost-effective assays for routine analysis of such drugs.

In comparison with conventional analytical techniques based on sophisticated, bulky, and costly instruments, chemical sensors, and in particular electrochemical sensors offer high sensitivity, high selectivity, fast analysis time, cost-effectiveness, eco-friendly, and handling ease. The merits of electrochemical sensors created a remarkably increased attention to these techniques, as well as research in this area is growing over the past decades.^32^ Even though, there are only two reports about electrochemical sensors for spiramycin. Unfortunately, both electrochemical sensors have limitations that may hinder their use in routine applications. The first reported electrochemical sensor for spiramycin is based on the use of a potentiometric polymer-membrane electrode.^22^ This potentiometric assay has the disadvantage of a very high limit of detection (6 × 10^−6^ M), which does not meet the spiramycin analysis requirements in real samples, consequently, it was only applied for the determination of SPA in its dosage forms.^22^ In addition, a such potentiometric sensor is based on the ion-exchange mechanism that is well-known for its low selectivity,^33^ because more lipophilic ions than the analyte are expected to exhibit high interference.^33^ The second electrochemical sensor for spiramycin was developed based on a carboxylic-multiwalled GCE modified with carbon nanotubes operated in the stripping linear sweep voltammetric mode (Ad-SLSV).^23^ This recently developed voltammetric sensor suffers from complicated fabrication procedures since it requires several chemical and electrochemical steps. The fabrication procedures require expensive chemicals/reagents and consume ample time.^23^ Moreover, this method has the disadvantage of using organic solvents (*e.g.*, dimethylformamide) which were crucial for the fabrication procedures. On the other hand, conventional voltammetric techniques (*e.g.*, polarographic methods^24^) are not suitable for SPA assay, because these methods used hanging mercury drop electrode, however, mercury is easily oxidized at the oxidation potential of SPA. Therefore, there is an urge for the development of a green, low-cost, less time-consuming and more sensitive electrochemical sensor for monitoring SPA in pharmaceutical formulations and biological fluids, as well.

GCE is one of the most significant electrodes used in electroanalytical techniques/sensors owing to its low cost, chemical stability, electrochemical inertness, high hardness, and ease of surface modification. Besides, GCE has a broad potential window in both anodic and cathodic regions.^34,35^ Depending on the nature of the redox system and the type of analysis, several activation procedures have been applied to obtain more reproducible results with a fast electron transfer^36^ which strongly relies on the prior history of pre-treatment. Different procedures have been reported for the pre-treatment of GCEs (*e.g.*, vacuum heating,^37^ treatment with laser,^38^ mechanical polishing,^39^ ultrasonication,^40^ and carbon arc^41^). Several electrochemical activation protocols have also been developed such as preanodization,^42–50^ preanodization followed by short-time cathodization, and precathodization.^36,51^ Among various activation protocols, electrochemical activation of GCE (AGCE) and its applications in the assay of drugs in their pharmaceutical formulations and/or biological fluids has gained quite great attention.^52^

Herein, this work aims to develop a green, simple, inexpensive, sensitive, selective, and fast voltammetric assay for SPA utilizing AGCE. One-step electrochemical activation of GCE was carried out by simple anodization at a high positive voltage (2.5 V). The developed AGCE-based SPA voltammetric sensor exhibited a low limit of detection, high selectivity, and high sensitivity. AGCE was utilized in the assay of SPA in the DPV mode. And the analytical utility of the developed method was demonstrated by the assay of SPA in spiked urine and milk samples, as well as pharmaceutical preparations (see below).

## Experimental

### Reagents

All chemicals were of analytical grade and used as received without further purification. SPA was kindly supplied by Atco Pharma for Pharmaceutical Industries Co. (Cairo, Egypt) and was used without additional purification. Spiramycin 150® was supplied by Atco Pharma for Pharmaceutical Industries Co., (Cairo, Egypt) and contains 42.8 g SPA per 100 g packet (batch # 161057). K_3_[Fe(CN)_6_] and K_4_[Fe(CN)_6_] were purchased from Oxford laboratory chemicals (India). NaCl and KNO_3_ were purchased from Koch-Light laboratory chemicals Ltd. (Colnbrook, England). KCl was purchased from BDH laboratory chemicals Ltd. (Poole, England). Glucose and lactose were purchased from Adwic laboratory. Phosphate buffer (pH, 7.0) was prepared from Na_2_HPO_4_ (0.1 M) and NaH_2_PO_4_ (0.1 M) and employed as the supporting electrolyte. Milk samples were purchased from local markets. Urine samples were kindly supplied by healthy human volunteers. Informed consents were obtained from the participants in our study.

### Instruments

Interface 1000 Gamry electrochemical workstation was used for performing all electrochemical preparations and measurements. A conventional three-electrode system was utilized. Bare GCE (Bioanalytical Systems, West Lafayette, IN, USA) model MF-2012 (3.0 mm in diameter) or AGCE was employed as the working electrode. Saturated double junction Ag/AgCl was used as the reference electrode while a platinum wire was utilized as the counter electrode. All experiments were carried out at a temperature of 25 ± 1 °C.

### Preparation of AGCE

**Fig. 1:**
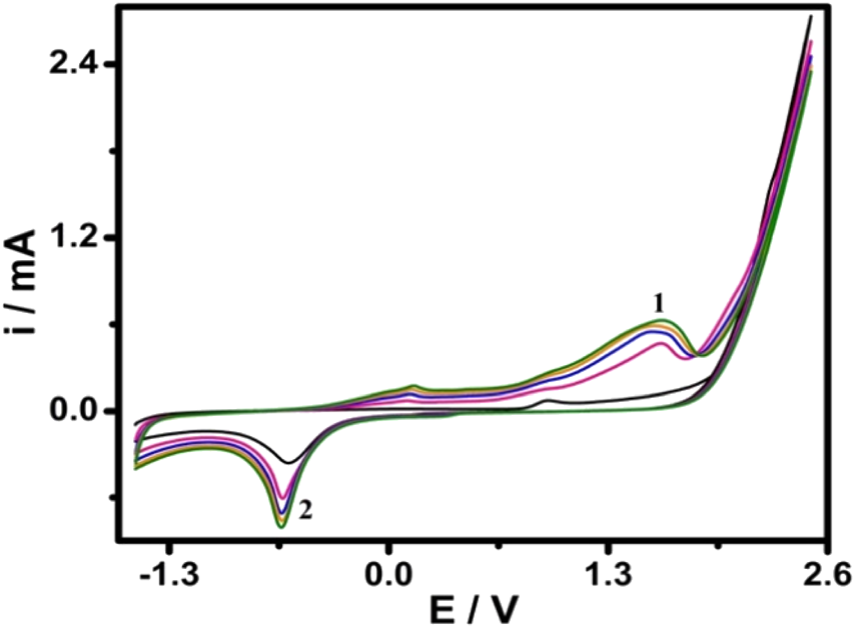
Repetitive cyclic voltammograms of GCE in 0.1 M phosphate buffer (pH 7.0). Scan rate: 100 mV s^−1^.

Prior to electrochemical activation, polishing of GCE (3 mm) diameter was performed using alumina slurry (0.05 μm) on a polishing micro cloth followed by thoroughly rinsing with ethanol and double distilled water to obtain a clean surface. To remove residues from the surface of the electrode, GCE was cleaned in an ultrasonic bath in double-distilled water for 2 min followed by 2 min in ethanol. GCE was electrochemically activated by immersing the bare GCE in 0.1 M phosphate buffer (pH 7.0) and conditioning the electrode by CV in the potential range (−1.5 to +2.5 V) at 100 mV s^−1^ for 5 scans (Fig. 1). The resulted electrode was designated as AGCE. Consequently, the AGCE was rinsed with double distilled water; afterward, the activated electrode was stored in phosphate buffer, pH 7.0, for later use.

### Preparation of stock standard solution

A stock standard solution of 0.1 M SPA was prepared by dissolving 9.9 g of pure drug in a 100 mL volumetric flask and completed to the mark with double distilled water. The working standard solution (5.0 × 10^−5^ M) was prepared daily by dilution of the stock standard solution with double distilled water.

### General analytical procedure

Into a series of 25-mL volumetric flasks, aliquots of the working standard solution equivalent to 3.1 × 10^−4^ to 8.0 × 10^−8^ M SPA were transferred, then the volume was completed with 0.1 M phosphate buffer (pH 7.0) and mixed well.

## Results and discussion

### Electrochemical activation of GCE

Cyclic voltammetry was utilized for the electrochemical activation of GCE. We have previously shown that CVs of GCE at a high anodic potential (2.5 V) in presence of organic monomer did not lead to electrochemical polymerization in many cases. Instead, this process resulted in the activation of GCE through the formation of surface functional groups (SFGs).^50^ Activated GCE, though, was proven to be responsible for obtaining useful responses towards various redox analytes.

As shown in Fig. 1, anodic peak 1 and cathodic peak 2 in the first scan were observed at ∼+1.5 V and −0.65 V, respectively. Larger peaks were observed upon continuous scanning, reflecting the continuous activation of GCE and the formation of SFGs on the surface of GCE after activation.^50^ The formed SFGs are subsequently reduced in the well-characterized cathodic process.^50^ The surface features of the AGC electrode were characterized as previously described in our work^50^ by scanning electron microscope, energy-dispersive X-ray spectrometer (EDX), Raman spectroscopy, and ATR-FTIR. We concluded, in our previous report,^50^ that the process of electrochemical activation led to forming of SFGs containing oxygen which have been determined using spectroscopic techniques (*e.g.*, EDX, Raman, and ATR-FTIR spectroscopy). Such an electrochemical activation process resulted in increasing the surface area^50^ and the formation of SFGs. Both factors facilitate electron transfer to/from target analytes at the surface of AGCE resulting in the improvement of electron transfer kinetic and the enhancement in the electrocatalytic activity. Enhancement of electron transfer is one of the quite important advantages of the electrochemical sensor which is attributed to the formation of SFGs and increasing the AGCE effective surface area. Herein, we demonstrate the analytical applications of the simple and one-step electrochemically activated glassy carbon electrode in the analysis of spiramycin.

### Electrocatalytic behavior of SPA at AGCE

**Fig. 2:**
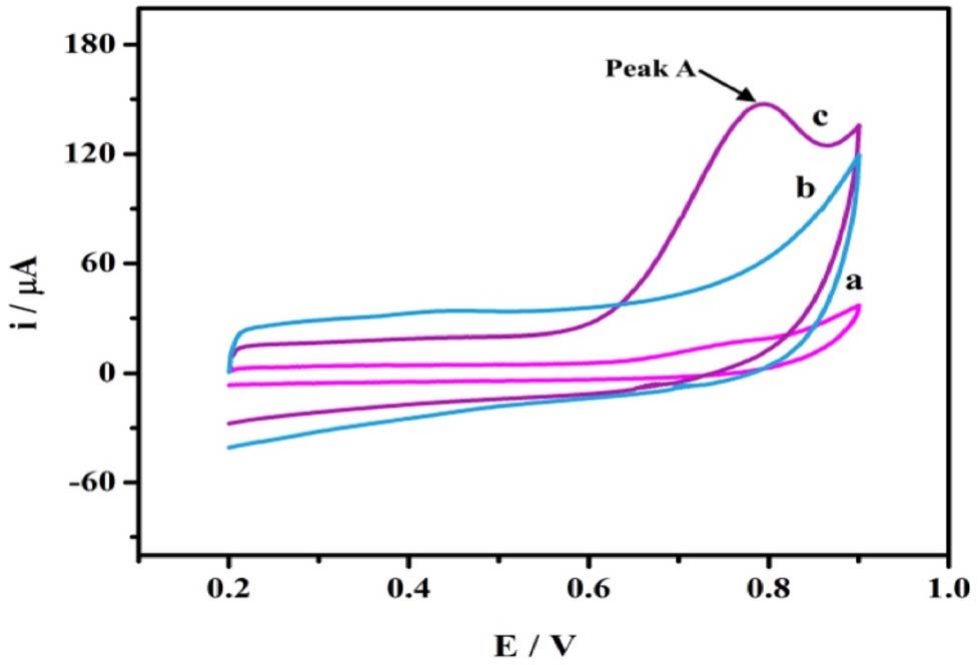
CVs of 1.0 × 10^−3^ M SPA in 0.1 M phosphate buffer pH 7.0 at bare GCE (a), and AGCE (c). Curve (b) represents the CV of AGCE in a blank solution (0.1 M phosphate buffer-free from SPA). Scan rate: 100 mV s^−1^.

**Scheme 1:**
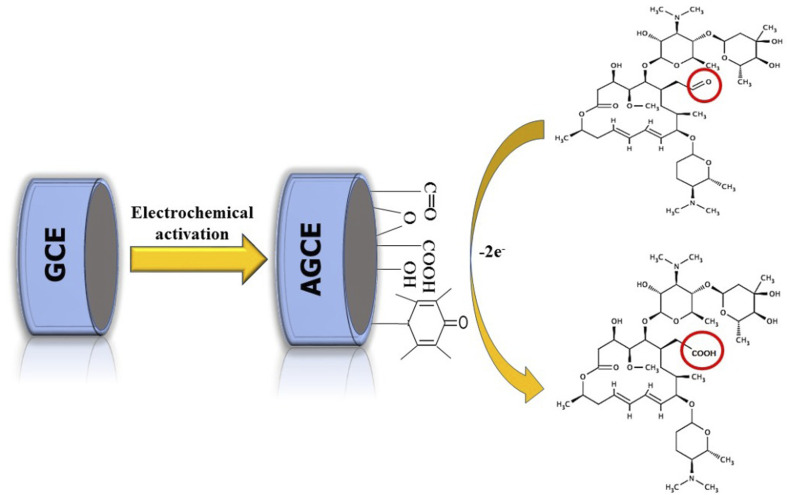
A schematic presentation of the possible electrooxidation mechanism of SPA at AGCE.

The electrochemical behavior of the SPA at AGCE was studied using CV in a potential window of 0.3 to 1 V. SPA (1.0 × 10^−3^ M) in phosphate buffer pH 7.0. As shown in Fig. 2 (curve a) an irreversible and weak oxidation peak at bare GCE is observed. A remarkable enhancement in oxidation peak current, however, is obtained for SPA at AGCE (Fig. 2, curve c). The 7-fold enhancement of peak current (peak A) observed at AGCE compared to bare GCE indicates that AGCE exhibits a remarkable enhancement effect for SPA electrocatalytic oxidation. This enhancement could be attributed to effective surface area enlargement, generation of redox-active sites (*i.e.*, oxygen-containing SF), increase in the ability of electron transfer, and high electrocatalytic activity of the AGCE toward SPA oxidation. To ensure that peak (A) corresponds to the oxidation of SPA, the performance of AGCE in a blank solution (*i.e.*, SPA free) was measured (Fig. 2, curve b). In the absence of SPA, peak (A) disappeared which declares its correlation to SPA oxidation. The role of the surface functional groups (SFGs) in the oxidation processes that occur at the surface of AGCE could be explained by the participation of the surface quinones, epoxides, and ketonic groups in the oxidation of the SPA-ketonic group to carboxylic group as depicted in Scheme 1.^50,53^

### Optimization of electrochemical conditions

The influences of various parameters, for example, accumulation time, scan rate, and pH of supporting electrolyte on the electrochemical response of AGCE towards SPA were studied and optimized. The AGCE response towards SPA was found to be significantly affected by those parameters.

### Effect of accumulation time

**Fig. 3:**
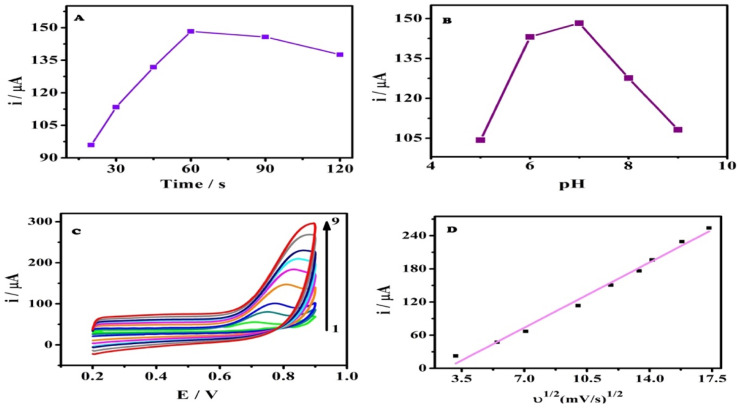
(A) Effect of accumulation time on the CV oxidation peak currents of 5.0 × 10^−5^ M SPA at AGCE. (B) Effect of solution pH on the CV oxidation peak currents of 5.0 × 10^−5^ M SPA at AGCE. (C) CVs of the AGCE at different scan rates in 0.1 M phosphate buffer (pH 7.0) containing 5.0 × 10^−5^ M SPA, scan rates (from 1 to 9): 10, 30, 50, 100, 140, 180, 200, 250 and 300 mV s. (D) A plot of peak current (*i*_p_) *versus* the square root of the scan rate (*ν*^1/2^).

Accumulation of spiramycin on the surface of the AGCE was investigated under open circuit potential for various time intervals. Afterward, cyclic voltammograms were recorded, Fig. S1.†Fig. 3A shows the influence of accumulation time on the oxidation peak current of SPA at the AGCE surface. There is a remarkable increase in the oxidation peak current from 20 to 60 s that reflects the enhancement of SPA adsorption on the AGCE surface, as can be seen in Fig. 3A. Nevertheless, with any further increase in the accumulation time, the current practically reached a plateau. This behavior might indicate that the active sites on the surface of the activated electrode have become saturated by SPA molecules. Considering work efficiency and sensitivity, 60 s was employed all through the study as the optimal accumulation time.

### Effect of solution pH

The influence of the pH of the sample solution on the electrochemical performance of AGCE towards SPA oxidation in 0.1 M phosphate buffer was investigated, over the pH range of 5.0 to 9.0, using the CV technique, as shown in Fig. S2.† The oxidation peak current was considerably affected by the solution pH, where the CV oxidation peak current increased by increasing the pH from pH 5.0 to pH 7.0. However, the peak current reached its maximum at pH 7.0 (Fig. 3B). While the current showed a remarkable sharp decrease upon any further increase in the pH of the solution. SPA has a p*K*_a_ of 7.9, which could account for the observed pH effect. At acidic pH, the SPA is positively charged, which facilitates its adsorption to the surface AGCE. Acidic oxygen-containing SFGs (*e.g.*, carboxylate) are expected to be negatively charged at slightly acidic or alkaline pH. At high pH values, however, it is expected that the adsorption of the SPA at AGCE is diminished because the SPA becomes neutral, which limits its adsorption to the negatively charged AGCE surface.

### Effect of varying scan rates

Cyclic voltammograms of a constant concentration of SPA (5 × 10^−5^ M) at different scan rates (*ν*) were recorded at the AGCE in 0.1 M phosphate buffer, pH 7 to investigate the effect of scan rate. Fig. 3C demonstrated that the oxidation peak current increased with the increase in the scan rate (*ν*). It can be also observed that the peak potential (*E*_p_) showed a slight shift with increasing scan rate. A plot of the oxidation peak current (*i*_p_) against (*ν*^1/2^) resulted in a linear relationship and a slope of 16.88 (*r*^2^ = 0.989), which indicates that the oxidation of SPA at the AGCE is a diffusion-controlled process (see Fig. 3D). This finding seems to be inconsistent with the adsorptive accumulation of SPA at the surface AGCE, however, the diffusion-controlled process could be related to SPA diffusion into AGCE open structure to reach the activated sites.^54–58^ To get a better understanding of the oxidation process of SPA at the surface of AGCE, a relationship of (log i_p_) to (log *ν*) was plotted (Fig. S3†). It is reported that the electrode process nature is reflected by the slope of this linear relationship as follows: a slope of 0.5 indicates diffusion-controlled,^59^ a slope of 1.0 indicates adsorption-controlled,^60^ and a slope between 0.5 and 1.0 indicates diffusion and adsorption controlled.^59^ Fig. S3† demonstrates that the linear relationship of (log i_p_) *versus* (log *ν*) exhibited a linear regression equation of log i_p_ (μA) = 0.61 + 0.73 log *ν* (mV s^−1^) (*r*^2^ = 0.999). The obtained slope, 0.73, indicates diffusion and adsorption-controlled electrode processes.

## The analytical performance of AGCE toward SPA

### Linearity range and LOD

**Fig. 4:**
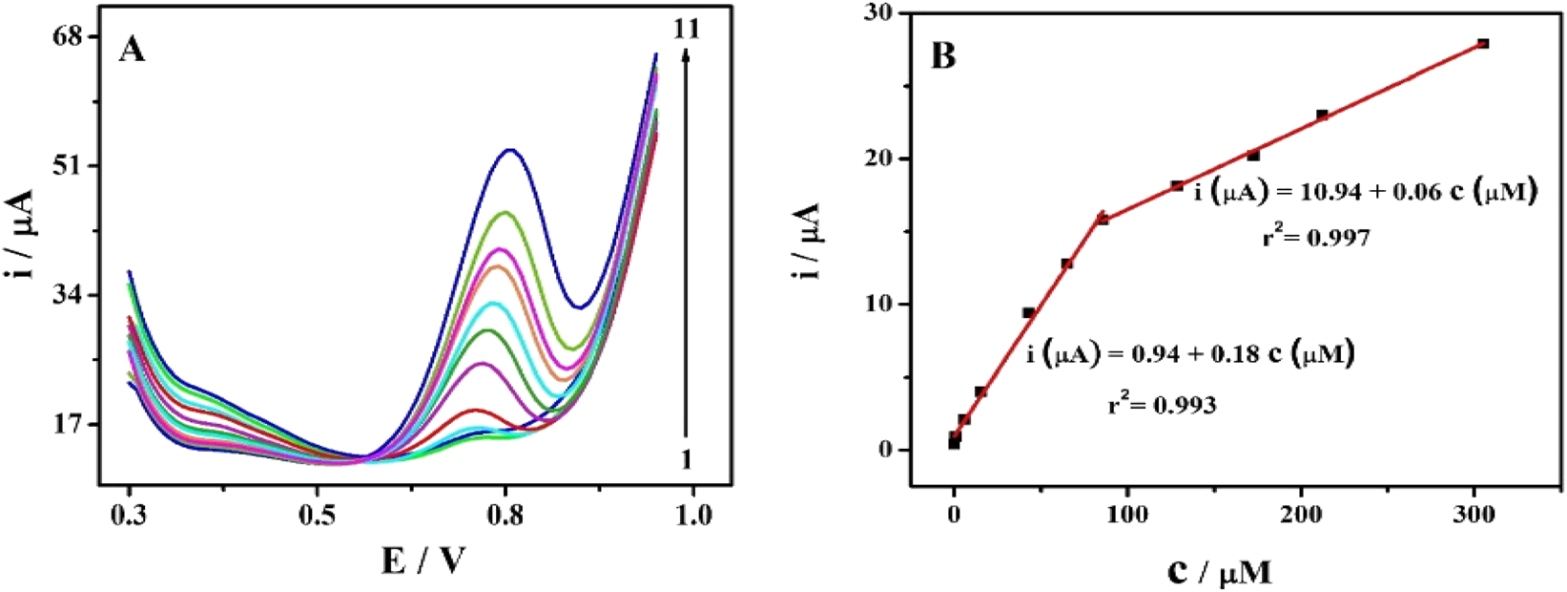
(A) DPVs at the AGCE in 0.1 M phosphate buffer (pH 7.0) containing different concentrations of SPA, (from 1 to 11): 0.08, 0.8, 5.9, 15.4, 43.0, 64.9, 85.8, 128.6, 172.6, 212.4 and 305.2 μM at scan rate: 100 mV s^−1^. (B) The corresponding calibration plot for SPA at AGCE.

The effect of increasing SPA concentration on the oxidation peak currents was investigated using cyclic voltammetric technique (Fig. S4†). The oxidation peak currents increased with increasing SPA concentrations as depicted in Fig. S4.† However, DPV is more sensitive and accurate, therefore, DPVs were recorded to study the influence of increasing SPA concentration on oxidation peaks of SPA. Under the optimized conditions, the previously mentioned analytical procedures were applied to eleven sets (three replicates for each concentration) of the studied drug. The AGCE revealed an excellent linear response for SPA in the investigated concentration ranges of 8.0 × 10^−8^ to 8.5 × 10^−5^ M and 8.5 × 10^−5^ to 3.1 × 10^−4^ M (see data represented in Fig. 4). The corresponding calibration plots showed a linear relationship with linear regression equations of i_p_ (μA) = 0.94 (±0.27) + 0.18 (±0.01) *c* (μM) (*r*^2^ = 0.993) and i_p_ (μA) = 10.94 (±0.29) + 0.06 (±0.001) *c* (μM) (*r*^2^ = 0.997). As shown in Fig. 4, the corresponding calibration plot displays two linear responses. This behavior could be explained based on the different diffusion rates of reaction products under the different spiramycin concentrations. At low SPA concentrations, the oxidation products can quickly leave away from the surface of the electrode. Nevertheless, the high concentrations of SPA obstruct the diffusion of the oxidation products. As a result, a lower oxidation peak current is detected in the high concentration range and a relatively higher oxidation current existed in the low concentration of SPA.^61^ This explanation is supported by the fact that the slope at lower concentrations of spiramycin (0.18) is three-fold higher than that at higher concentrations (0.06). The limit of detection was calculated according to IUPAC regulations,^62^ LOD = 3.3 × *σ*/*S*, where *σ* is the standard deviation of the blank and *S* is the slope of the calibration curve. The limit of detection (LOD) was estimated to be 2.0 × 10^−8^ M, which is 10-fold lower than the MRLs of spiramycin.^13^

### Repeatability, stability, and selectivity studies

The repeatability of AGCE was investigated using intra- and interday precisions. The intraday precision of the proposed sensor was studied by performing 10 replicate measurements in 5.0 × 10^−5^ M SPA using the same AGCE. As a result, the relative standard deviation (RSD) was estimated to be 0.99%, showing excellent repeatability of the activated electrode (Fig. S5†). Additionally, the interday precision was evaluated by detecting 5.0 × 10^−5^ M of the studied drug in duplicate with three AGCEs prepared exactly independently. The RSD for such measurements was estimated to be 1.3%, Fig. S6.† Thus, the developed sensor offers excellent precision for SPA determinations.

The AGCE stability was examined by recording the current responses at a fixed SPA concentration (5.0 × 10^−5^ M) for 10 days. Between measurements, the modified electrode was kept at room temperature (25 °C) in 0.1 M phosphate buffer, pH 7. As shown in Fig. S7,† the optimized AGCE current response remained stable for one week, after which, the current response decreased by 8.79%, which confirms the reasonable stability of the AGCE. It is worth mentioning that regeneration requires neither applying complicated procedures nor the use of expensive material,^23^ but merely repeating the electrochemical activation procedures described above.

Additionally, the AGCE selectivity was investigated. The interference criterion is dependent on the current response. If the addition of a specific species causes an alteration in the current with 5% or more, then it is considered to cause interference.^63^ Various foreign species were added into 0.1 M phosphate buffer (pH 7.0) containing 5.0 × 10^−5^ M SPA to investigate any possible interferences for the monitoring of SPA at the modified sensor. The tolerance level, which causes less than 5% change in the current was found to be 5000-fold for Na^+^, K^+^, Cl^−^, NO_3_^−^, glucose, and lactose, and 10-folds for spectinomycin hydrochloride, see Fig. S8.† This reveals that the optimized AGCE has high tolerance level against glucose, lactose, most common ions, and a representative veterinary drug (see data depicted in Table S1†).

## Application of AGCE

### Assay of SPA in its pharmaceutical dosage form

The commercial dosage form of SPA was analyzed using the developed protocol and the reported procedure,^19^ as well. In each case, three replicate measurements were made and the data obtained using the AGCE were statistically compared with the reported procedure.^19^ The mean percentage recoveries were estimated to be 99.7 ± 1.01 (Table 1). Comparison of the analysis results of the suggested and the reported methods, at a 95% confidence level with respect to *t* and *F*-tests,^64,65^ indicated that there are no significant variations between calculated and theoretical values. This reflected that the developed method has excellent accuracy and precision in the analysis of the pharmaceutical formulation.

**Table 1:** Determination of SPA in pharmaceutical formulation using AGCE and the reported methods

Dosage form	% recovery^a^ ± SD		*t*-value^b^	*F*-value^a^
	Proposed method	Reported method^19^		
Spiramycin 150®	99.7 ± 1.01	99.3 ± 0.78	1.22	2.38

a The value is the average of five determinations.

b The tabulated values at 95% confidence limits are *t* = 2.306 and *F* = 6.338.

### Assay of SPA in spiked milk and urine samples

The analytical utility of the developed AGCE in the assay of SPA was further proven by the analysis of SPA in real samples (*e.g.*, milk samples available in local markets) and in biological samples (*e.g.*, urine from healthy human volunteers). To ensure the absence of SPA in the milk and urine samples as well, non-spiked samples were analyzed using the developed method and no SPA-related peaks were observed. Then milk and urine samples were spiked to different concentrations of SPA and the spiked concentrations were measured using AGCE and the recoveries were calculated. Results obtained indicated that the suggested protocol was successfully applied for the accurate assay of spiked SPA in milk and urine samples as indicated by the observed recoveries of 98.1–102% and 98.4 to 101%, for milk and urine samples, respectively, at different spiked concentrations (Table 2). The standard deviation (SD) of the obtained recoveries was in the range of (0.1–0.3) and (0.1–0.4) for milk and urine samples respectively (see data depicted in Table 2).

**Table 2:** Determination of SPA in spiked milk and urine samples using AGCE

Sample	Milk			Urine		
	Added, μM	Mean concentration found^a^ by AGCE, μM	% recovery ± SD	Added, μM	Mean concentration found^a^ by AGCE, μM	% recovery ± SD
1	5.0	5.09	102.0 ± 0.1	5.0	4.92	98.4 ± 0.1
2	10.0	9.94	99.4 ± 0.1	10	9.86	98.6 ± 0.4
3	20.0	19.6	98.1 ± 0.3	20	20.01	100.0 ± 0.4
4	40.0	39.2	98.1 ± 0.2	40	40.3	101.0 ± 0.4
Mean ± SD			99 ± 2			100 ± 1
% RSD			2			1

a Average of three determinations.

## Comparison of the proposed method with different analytical techniques for the determination of SPA

In comparison with various methods reported in the literature for the analysis of SPA, the developed simple and one-step electrochemically activated GCE exhibited a low limit of detection, and a wide range of linearity better than most of the reported methods of analysis (Table 3). As depicted in Table 3, the supposed AGCE-based SPA sensor exhibited superior analytical performance compared to the reported potentiometric sensor^22^ and the polarographic assay^24^ in terms of less time-consuming, fewer pre-treatment steps, and wider linear ranges (8.0 × 10^−8^ to 8.5 × 10^−5^ M and 8.5 × 10^−5^ to 3.1 × 10^−4^ M). The modified electrode exhibited a limit of detection (2.0 × 10^−8^ M) that is ∼300-fold lower than the potentiometric method (5.9 × 10^−6^ M), and ∼40, 500-fold lower than DPP and SWP, respectively. Compared with the recently developed Ad-SLSV assay,^23^ the proposed sensor for SPA exhibited wider linear ranges (8.0 × 10^−8^ to 8.5 × 10^−5^ M and 8.5 × 10^−5^ to 3.1 × 10^−4^ M), a lower limit of detection (2.0 × 10^−8^ M). The AD-SLSV assay of the studied drug,^23^ however, exhibited ∼5-fold better sensitivity compared to the developed AGCE-based SPA electrode. The RP-HPLC-fluorescence method^12^ showed a limit of the linear range (5.9 × 10^−8^ M) lower than the modified electrode (8.0 × 10^−8^ M). On the other hand, the proposed AGCE does not require expensive materials or complicated instrumentation and does not involve complicated fabrication procedures. Furthermore, the proposed AGCE sensor for SPA is characterized by excellent selectivity, repeatability, stability, and remarkable fabrication simplicity. Additionally, this protocol is an eco-friendly, reliable, and cost-effective method of analysis for monitoring SPA in its dosage form and real samples.

**Table 3:** Analytical features of different analytical techniques for the detection of SPA^a^^,^^b^

Method	Linear range (M)	LOD (M)	Reference
RP-HPLC-fluorescence	5.9 × 10^−8^ to 5.9 × 10^−7^	5.9 × 10^−6^	12
RP-HPLC-UV	5.9 × 10^−8^ to 2.4 × 10^−5^	NR	14
Solid phase extraction and HPLC-UV	1.2 × 10^−7^ to 1.2 × 10^−6^	5.9 × 10^−8^	15
	1.2 × 10^−6^ to 1.2 × 10^−5^		
RP-HPLC-DAD	1.2 × 10^−6^ to 2.4 × 10^−5^	1.3 × 10^−7^	16
HPLC-UV	3.6 × 10^−7^ to 1.4 × 10^−3^	NR	17
RP-HPLC-UV	4.8 × 10^−6^ to 5.9 × 10^−3^	1.8 × 10^−6^	18
Spectrophotometry	5.9 × 10^−6^ to 2.9 × 10^−5^	3.6 × 10^−7^	19
LC	5.9 × 10^−4^ to 1.8 × 10^−3^	3.0 × 10^−7^	20
CE	1.0 × 10^−7^ to 1.0 × 10^−5^	7.5 × 10^−8^	21
Potentiometry	1.0 × 10^−5^ to 1.0 × 10^−2^	5.9 × 10^−6^	22
Ad-SLSV	1.0 × 10^−7^ to 4 × 10^−5^	2.8 × 10^−8^	23
DPP	2.4 × 10^−5^ to 9.5 × 10^−5^	1.0 × 10^−5^	24
	9.5 × 10^−5^ to 1.1 × 10^−5^		
SWP	1.2 × 10^−5^ to 9.5 × 10^−5^	7.6 × 10^−7^	
DPV	8.0 × 10^−8^ to 8.5 × 10^−5^	2.0 × 10^−8^	This work
	8.5 × 10^−5^ to 3.1 × 10^−4^		

a NR: not reported.

b UV: ultraviolet; DAD: photodiode array detector; LC: liquid chromatography; CE: capillary electrophoresis; Ad-SLSV: adsorptive stripping linear sweep voltammetry; DPP: differential pulse polarography; SWP: square wave polarography. The electroactive material for AD-SLSV: carboxylic multiwalled glassy carbon electrode modified with carbon nanotubes, DPP, and SWP: hanging mercury drop electrode.

## Conclusion

GCE pretreated with a simple anodization procedure at 2.5 V was utilized for the selective, and sensitive, determination of SPA using DPV. The electrochemical AGCEs were studied using cyclic voltammetry (CV) that indicated a significant increase in the anodic peak of SPA in comparison with the bare GCE. The developed sensor can be used for routine analysis of SPA in real and biological samples owing to its ease of fabrication, cost-effectiveness, high sensitivity, excellent repeatability, and low detection limit that meets SPA-analysis requirements. In comparison with the previously reported electrochemical methods, the developed sensor exhibited better performance in terms of linear ranges and LOD. Moreover, the developed AGCE is cost-effective, environmentally friendly, and offers operationally simple since there is no complicated modification procedure for the surface of the GCE. Under optimized conditions, the developed AGCE exhibited linear ranges of 8.0 × 10^−8^ to 8.5 × 10^−5^ M and 8.5 × 10^−5^ to 3.1 × 10^−4^ M with a detection limit as low as 20 nM. The developed AGCE-based sensor for SPA was applied for the determination of SPA in its pharmaceutical formulation and efficiently utilized to analyse SPA in different diluted urine and milk samples with satisfactory recovery results.

## Author contributions

The first, second, and last authors contributed to the study's conception and design. Data collection was performed by the first and second authors. Analysis of data was performed by the first second and third authors. The first draft of the manuscript was written by the first author. All authors read and approved the final manuscript.

## Conflicts of interest

There are no conflicts to declare.

## Supplementary Material

RA-013-D2RA06768D-s001
