# Supplementary material for: Green and cost-effective voltammetric assay for spiramycin based on activated glassy carbon electrode and its applications to urine and milk samples
Source: RSC Adv. 2023 Jan 3;13(2):844–52. doi: 10.1039/d2ra06768d (PMC9809205; doi:10.1039/d2ra06768d)
Supplement: RA-013-D2RA06768D-s001 [file RA-013-D2RA06768D-s001.pdf]

Electronic Supplementary Material (ESI) for RSC Advances.
This journal is © The Royal Society of Chemistry 2022

Supporting information for:

Green and cost-effective voltammetric assay for spiramycin based on activated glassy carbon electrode and its applications to urine and milk samples

Hind A. M. Noureldin[a], Ali M. Abdel-Aziz[b], Mokhtar M. Mabrouk[c], Amira H. K. Saad[c], Ibrahim H. A. Badr [b,d*]

[a] Department of Analytical Chemistry, Faculty of Pharmacy, Badr University in Cairo, Cairo, Egypt

[b] Department of Chemistry, Faculty of Science, Ain Shams University, Cairo, Egypt

[c] Department of Analytical Chemistry, Faculty of Pharmacy, Tanta University, Tanta, Egypt

[d] Department of Chemistry, Faculty of Science, Galala University, Suez, Egypt

[*] Corresponding author e-mail: ibrahim.badr@gu.edu.eg; ihbadr@sci.asu.edu.eg.

Table of Contents

Fig. S1. CVs of 5.0×10^{-5} M SPA at different accumulation times. (from 1 to 6): 20, 30, 45, 120, 90 and 60 s……………………………...……………………….……………......**S-3**

Fig. S2. CVs of 5.0×10^{-5} M SPA at different pH, (from a to e): pH 5, 6, 9, 8 and 7…....………………...……………………….....……………….……………......**S-4**

Fig. S3. A plot of logarithm peak current (log i_p) versus the logarithm of the scan rate (log υ)………....………………....……………………………..………………….......**S-5**

Fig. S4. CVs of SPA at different concentrations (from 1 to 6): 5.0×10^{-5}, 4.0×10^{-4}, 3.0×10^{-4}, 2.0×10^{-4}, 1.0×10^{-4} and 1.0×10^{-3} M SPA. Scan rate: 100 mV/s……………………..…. **S-6**

Fig. S5. The intraday precision of the AGCE ……………………………….… …….. **S-7**

Fig. S6. The interday precision of the AGCE …………………………………….…… **S-8**

Fig. S7. The lifetime of the AGCE ……………………….. ………………..……. **S-9**

Fig. S8. DPVs of the AGCE in 0.1 M phosphate buffer (pH 7.0) containing (a) 5.0×10^{-5} M SPA and different interferent concentrations (b) 0.1 and (c) 0.25 M. (A) NaCl, (B) KNO_3, (C) glucose, (D) lactose and (E) Spectinomycin sulfate……………………………..…. **S-10**

Table S1: Study the effect of different concentrations of foreign species on the oxidation peak current of 5.0×10^{-5} M SPA measured at AGCE…………..…………………..…**S-11**

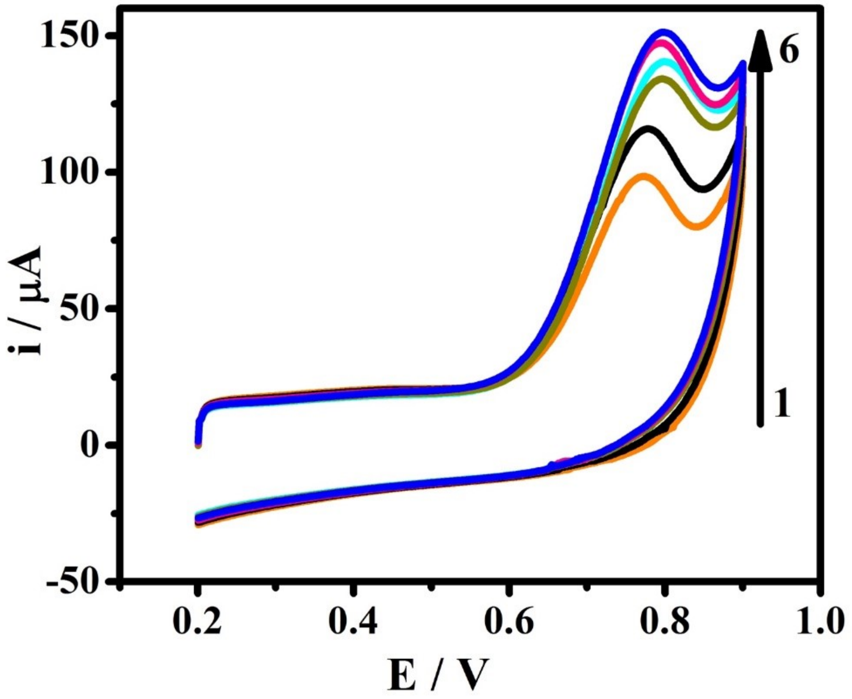

Fig. S1. CVs of 5.0×10^{-5} M SPA at different accumulation times. (from 1 to 6): 20, 30, 45, 120, 90, and 60 s.

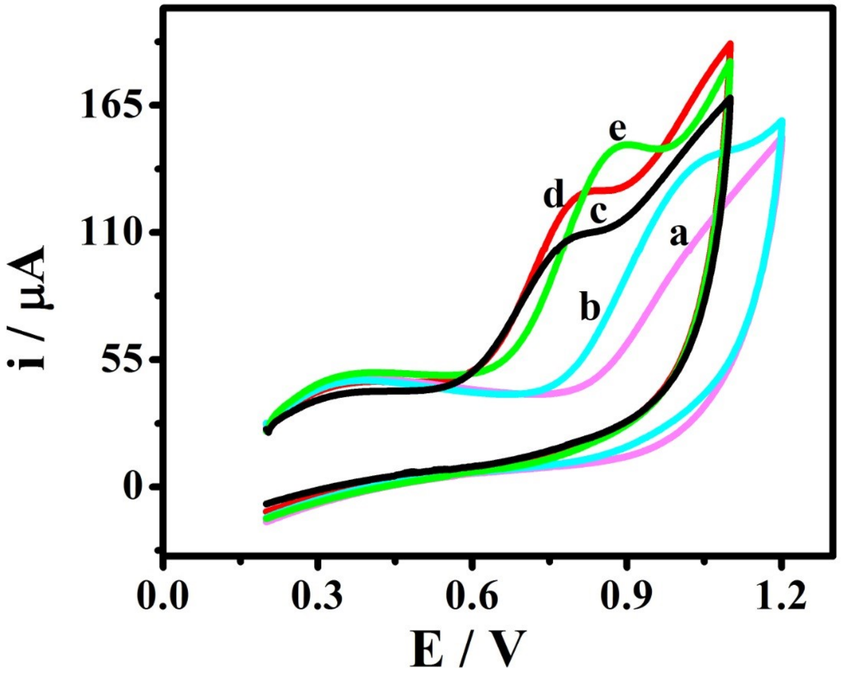

Fig. S2. CVs of 5.0×10^{-5} M SPA at different pH, (from a to e): pH 5, 6, 9, 8, and 7**.**

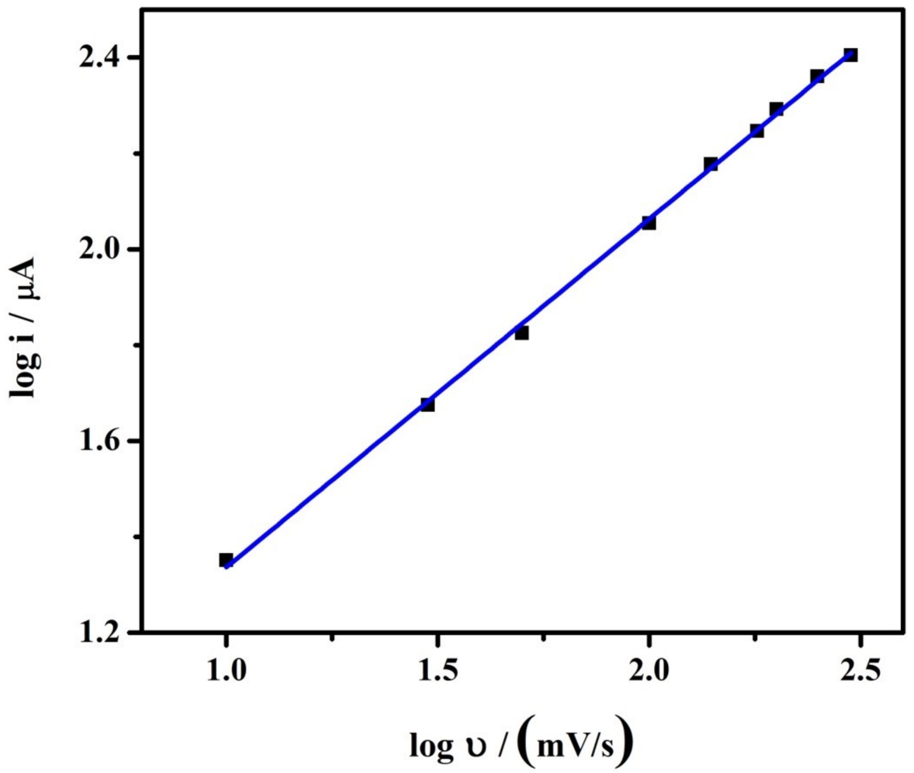

Fig. S3. A plot of logarithm peak current (log i_p) versus the logarithm of the scan rate (log υ).

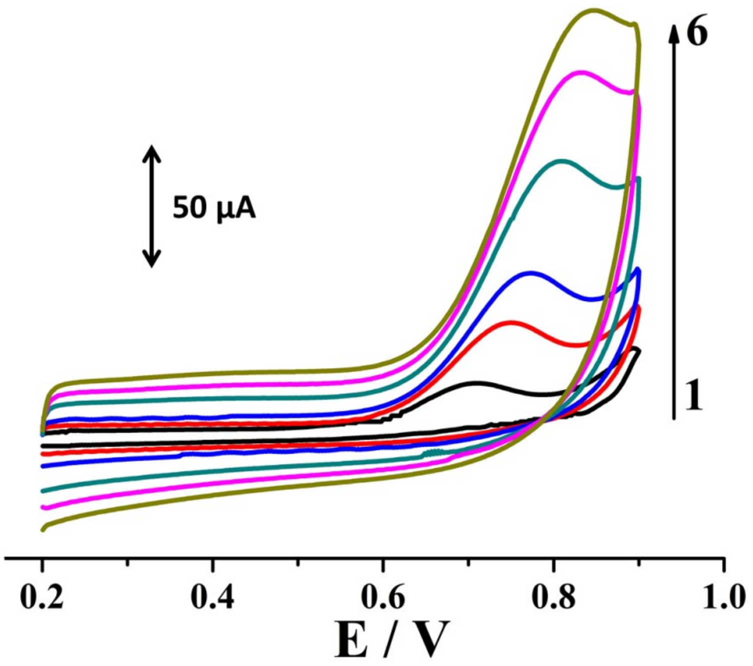

Fig. S4. CVs of SPA at different concentrations (from 1 to 6): 5.0×10^{-5}, 4.0×10^{-4}, 3.0×10^{-4}, 2.0×10^{-4}, 1.0×10^{-4} and 1.0×10^{-3} M SPA. Scan rate: 100 mV/s.

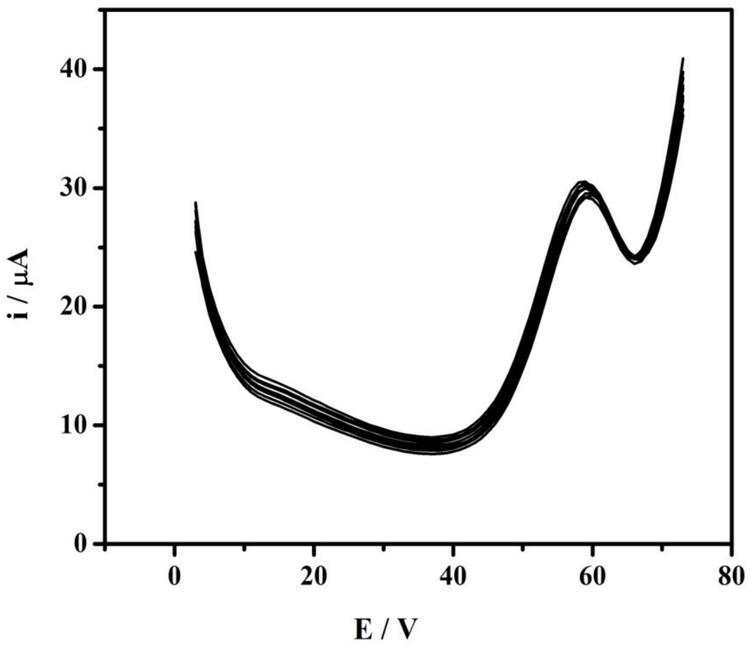

Fig. S5: The intraday precision of the AGCE.

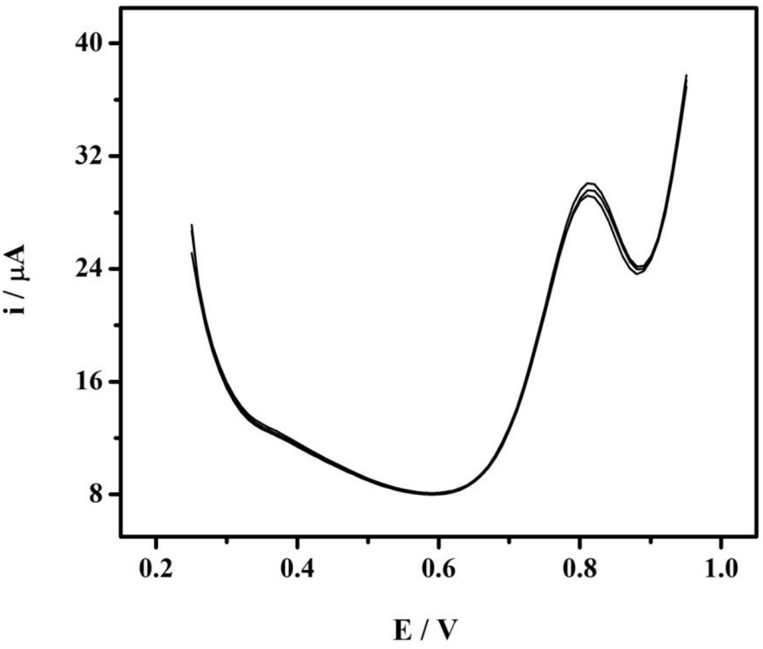

Fig. S6. The interday precision of the AGCE.

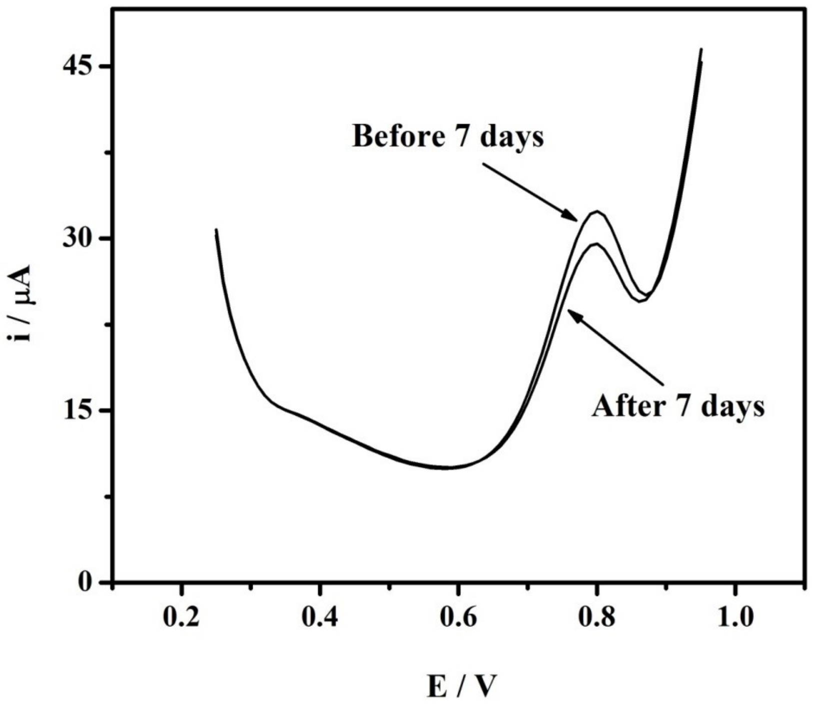

Fig. S7. The lifetime of the AGCE.

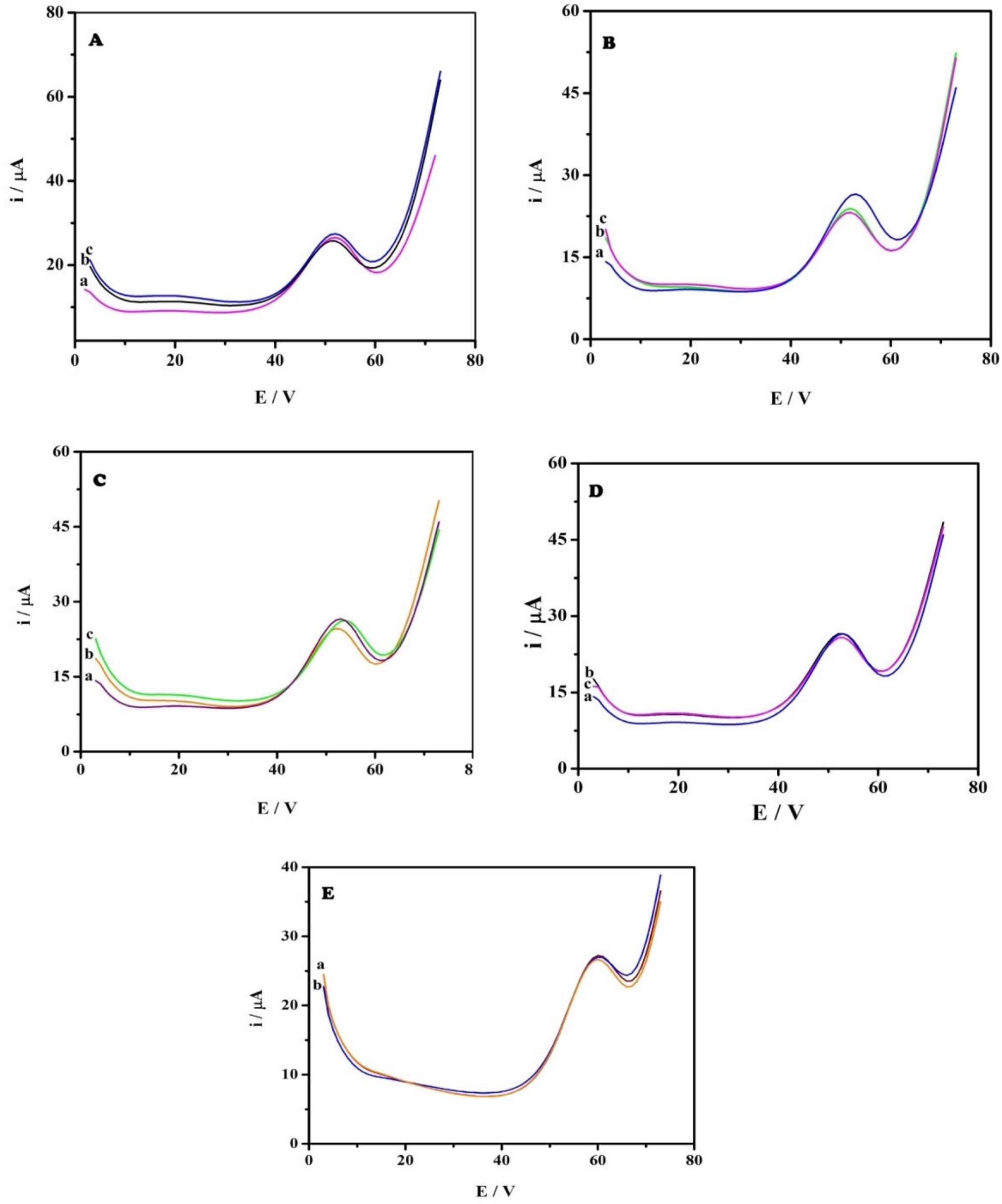

Fig. S8. DPVs of the AGCE in 0.1 M phosphate buffer (pH 7.0) containing (a) 5.0×10^{-5} M SPA and different interferent concentrations (b) 0.1 and (c) 0.25 M of (A) NaCl, (B) KNO_3, (C) glucose, (D) lactose and (E) Spectinomycin sulfate.

Table S1: Study the effect of different concentrations of foreign species on the oxidation peak current of 5.0×10^{-5} M SPA measured at AGCE.

Substance	Concentration (M)	Signal change (%)
Na^+	0.10	2.13
	0.25	2.77
K^+	0.10	0.850
	0.25	1.38
Cl^-	0.10	2.13
	0.25	2.77
NO_3^-	0.10	0.850
	0.25	1.38
Glucose	0.10	1.28
	0.25	3.51
Lactose	0.10	2.13
	0.25	3.93
Spectinomycin hydrochloride	0.0062	1.45
